# Adherence to National Comprehensive Cancer Network Guidelines for *BRCA* testing among high risk breast Cancer patients: a retrospective chart review study

**DOI:** 10.1186/s13053-020-00144-z

**Published:** 2020-06-06

**Authors:** Priyanka Bobbili, Temitope Olufade, Maral DerSarkissian, Rahul Shenolikar, Hong Yu, Mei Sheng Duh, Nadine Tung

**Affiliations:** 1 Analysis Group, Inc., 111 Huntington Avenue, 14th Floor, Boston, MA 02199 USA; 2 AstraZeneca, 950 Wind River Ln, Gaithersburg, MD 20878 USA; 3 Analysis Group, Inc., 333 South Hope Street, 27th Floor, Los Angeles, CA 90071 USA; 4 Beth Israel Deaconess Medical Center, 330 Brookline Ave, Boston, MA 02215 USA

**Keywords:** Breast cancer, *BRCA* variant, *BRCA* testing, NCCN guidelines, Genetic testing

## Abstract

**Background:**

Testing for *BRCA* variants can impact treatment decisions for breast cancer patients and affect surveillance and prevention strategies for both patients and their relatives. National Comprehensive Cancer Network (NCCN) guidelines recommend testing for patients at heightened risk of *BRCA* pathogenic variant. We examined the *BRCA* testing rate among high risk breast cancer patients treated in community oncology practices.

**Methods:**

We conducted a retrospective medical chart review among community-based US oncologists using a physician panel approach. High risk breast cancer patients with a known family history of cancer and diagnosis with breast cancer at age ≥ 18 years between January 2013–October 2017 were included. We assessed the proportions of patients tested for *BRCA* variants in accordance with NCCN guidelines.

**Results:**

Charts from 63 physicians, averaging 16 years of practice, were included; 97% were medical oncologists and 66.7% had a genetic counselor in their practice. We analyzed data for 410 randomly-selected patients with mean age of 52 years; 95% were female, 74% were White, and 19% had Ashkenazi Jewish ancestry. Among all patients, 94% were tested for *BRCA* variants. The testing rate ranged from 78 to 100% in various high risk groups; lower rates were observed among Black patients (91%), men (92%), and patients meeting NCCN criteria based on family history of male breast cancer (78%) and prostate cancer (87%). We observed a higher testing rate in patients treated by physicians with a genetic counselor in their practice (95% versus 91%).

**Conclusions:**

Adherence to NCCN *BRCA* testing guidelines is high in this group of predominantly medical oncologists with extensive experience, with a high proportion having a genetic counselor in practice. Testing rates can be improved in patients with risk factors related to male relatives. High level of compliance to guidelines in a community setting is possible with a delivery model for genetic counseling and testing.

## Background

Pathogenic variants in the tumor suppressor breast cancer genes *BRCA1* and *BRCA2* increase the risk of female breast and ovarian cancers. Approximately 57% of women with an inherited *BRCA1* pathogenic variant and 49% with a *BRCA2* pathogenic variant develop breast cancer by 70 years of age [1], compared to a lifetime risk of 12.5% in women in the general population [2].

Testing for *BRCA* variants may impact cancer prevention or treatment decisions in patients at heightened risk of hereditary breast cancer [3]. One study assessing 220 breast cancer patients reported a significantly higher proportion of contralateral prophylactic mastectomy, which may positively impact survival, in patients who were aware of their *BRCA* variant status compared to patients who were not aware (76.4% vs. 14.7%) [4]. Alternatively, identifying pathogenic variant carriers allows risk reducing salpingo-oophrectomy which has been shown to improve survival [5, 6]. Currently, most patients with breast or ovarian cancer receive chemotherapy or hormone therapy as first-line treatment, regardless of *BRCA* status. As new, targeted therapies for breast cancer such as poly (ADP-ribose) polymerase (PARP) inhibitors are approved for use, knowledge of *BRCA* status is essential for understanding optimal treatment options for breast cancer patients.

National Comprehensive Cancer Network (NCCN) guidelines recommend genetic counseling and testing for *BRCA1*/*BRCA2* status for patients at heightened risk of having pathogenic variants due to personal or family history. Testing for *BRCA* pathogenic variants has been available since 1996, and the rate of testing among breast cancer patients has been previously assessed. In 2005, Brown et al. reported that among 551 surveyed women with early onset breast cancer, only 45% had discussed genetic testing with their physicians and/or been referred to a genetic counselor; those with a family history of cancer (53%) and Ashkenazi Jewish women (81%) were more likely to have been referred to a genetic counselor [7]. Additionally, a 2011 study reported that only 29.1% of patients with breast cancer were referred for genetic counseling and/or testing by their physicians [8].

Despite the importance of *BRCA* testing for breast cancer treatment decisions, few recent studies have assessed the rate of testing among breast cancer patients at an increased risk for pathogenic variants. A study by Wood et al. found that, among breast cancer patients diagnosed between 2009 and 2011, 52.2% of patients with an increased risk for hereditary breast cancer were referred for genetic counseling and/or testing [8]. This proportion may have changed following 2013, due to expanded availability of *BRCA* testing. Understanding *BRCA1*/*BRCA2* testing rates in breast cancer patients at a higher risk of having pathogenic variants, especially those treated in a community oncology setting where testing rates have traditionally been lower than in academic settings [9], will help inform strategies to improving awareness and testing of *BRCA* in eligible patients. The present study assessed the proportion of recently diagnosed, high risk, breast cancer patients treated in a US community oncology setting, who were tested for *BRCA1*/*BRCA2* variants in accordance with NCCN guidelines. The proportion of tested patients who were positive for a *BRCA1* or *BRCA2* pathogenic or likely pathogenic variant was also assessed.

## Methods

### Data source, study design, and population

An online physician panel approach was used to recruit medical oncologists for this retrospective chart review study, conducted from June 2017 to April 2018. Cardinal Health Specialty Solutions sent open invitations to its panel of community-based oncologists who treat breast cancer in the US. Eligible oncologists were required to be able to participate in research approved by a central institutional review board (IRB) and to have treated at least one patient with breast cancer in the past year.

Oncologists who elected to participate in this study were blinded to the study sponsor and not informed of the study objective. They randomly selected patient charts based on a “random-letter” generating algorithm used to identify patient last names, to mitigate concerns about selection bias, and completed a web-based electronic case report form (eCRF) to collect secondary data from up to 10 breast cancer patients. To be eligible for this study, patients were required to have a confirmed diagnosis of breast cancer between January 2013 and October 2017 and an accompanying pathology report such that hormone receptor (HR) status (i.e., estrogen receptor [ER], progesterone receptor [PR]) and Human Epidermal Growth Factor Receptor 2 (HER2) status were known. They were required to be 18 years of age or older at initial breast cancer diagnosis, have information on family history of cancer, and have an increased risk of hereditary breast cancer. Criteria to identify increased hereditary risk were developed based on NCCN testing guidelines (as available from 2013 to 2017, across all years under study) and those commonly used to identify high risk patients in clinical practice (Additional file 1). Hereditary risks included personal and family history of breast cancer or other cancers, and Ashkenazi Jewish ancestry. Patient selection was designed to oversample triple negative breast cancer (TNBC) and metastatic breast cancer patients.

The primary outcomes of the study were *BRCA* testing status and results of testing. In addition, data on physician characteristics (e.g., primary medical specialty, practice setting and size, presence of a genetic counselor in their practice, and familiarity with NCCN guidelines) and patient characteristics (e.g., demographic information, breast cancer pathology, and personal and family history of breast and other cancers) were also collected through the eCRF.

### Statistical analysis

Descriptive analyses were conducted to characterize physicians who participated in the study and to summarize demographic and clinical information for breast cancer patients meeting eligibility criteria, for whom data were collected.

The proportion of high risk patients who were tested in accordance with NCCN *BRCA* testing guidelines was calculated overall for the total study population and for risk groups defined based on NCCN testing guidelines released during the study period (2013–2017) (Additional file 1). Patients could belong to more than one high risk group. Stratified analyses by year were conducted to assess trends over the study period. The proportion of these patients who tested positive for a *BRCA1*/*BRCA2* pathogenic variant was also assessed in each risk group.

Stratified analyses were performed in patients with metastatic and triple negative breast cancer, patients with metastatic and HER2 negative (−) breast cancer, African American patients, patients treated in practices with a genetic counselor, and patients treated in practices without a genetic counselor.

All analyses were conducted in SAS 9.4 (SAS Institute, Cary, N.C.).

## Results

### Physician and patient characteristics

A total of 63 oncologists elected to participate in this study, of whom the majority were general oncologists (97%). The participating oncologists had, on average, 16 years of practice and were proportionally distributed across the Northeast, Midwest, South, and West. They reported treating an average of 154 breast cancer patients in the previous year. All oncologists indicated familiarity with NCCN *BRCA* guidelines, and 67% had a genetic counselor as part of their practice (Table 1).

**Table 1:** Physician and Practice Characteristics

	Physicians
	(*N* = 63)
**Physician information**	
**Specialty, n (%)**	
General Oncology	61 (96.8)
Radiation Oncology	2 (3.2)
**Years of practice, mean (SD) [median]**	16 (6.6) [15.0]
**Breast cancer patients personally managed in the past year, mean (SD) [median]**	154 (171.7) [100.0]
**Familiar with NCCN*BRCA*guidelines, n (%)**	
Yes	63 (100.0)
**Practice information**	
**Practice setting, n (%)**	
Solo practitioner	4 (6.3)
Small private community practice (2–5 physicians)	13 (20.6)
Medium-sized private community practice (6–10 physicians)	16 (25.4)
Large private community practice (> 10 physicians)	16 (25.4)
Community practice owned by a hospital	12 (19.0)
Other	2 (3.2)
**Region**^a^**, n (%)**	
Northeast	17 (27.0)
Midwest	12 (19.0)
South	17 (27.0)
West	17 (27.0)
**Genetic counselor in practice, n (%)**	
Yes	42 (66.7)
No	21 (33.3)
**Method for genetic counselling**	
Referral to genetic counselling program	15 (71.4)
Referral to genetic testing company	3 (14.3)
Referral to genetic counselor telephone line	1 (4.8)
Other	2 (9.5)

***Abbreviations*:***BRCA* Breast Cancer Gene, *NCCN* National Comprehensive Cancer Network, *SD* Standard Deviation

^a^ Regions defined as: Northeast - CT, DE, MA, ME, MD, NH, NJ, NY, PA, RI, and VT; Midwest - IA, IL, IN, KS, MI, MN, MO, ND, NE, OH, SD, and WI; South - AR, AL, DC, GA, FL, KY, LA, MS, NC, OK, SC, TN, TX, VA, and WV; West - AK, AZ, CA, CO, ID, HI, MT, NM, NV, OR, UT, WA, and WY.

Among 410 patients included in the study, the mean age at breast cancer diagnosis was 52 years, the majority (95%) were female, White (74%), and 19% were of Ashkenazi Jewish ancestry (Table 2). At diagnosis, 126 patients (31%) had TNBC and 124 patients (30%) had metastatic breast cancer, of whom 105 (85%) were HER2(−) and 34 (27%) were triple negative. Patients with metastatic and triple negative breast cancer appeared to have a lower mean age at diagnosis (51 vs. 57 years) and were less likely to have been previously treated for ovarian cancer (3% vs. 5%) compared to patients with metastatic and HER2(−) breast cancer (Additional file 1).

**Table 2:** Demographic and Clinical Characteristics in All Patients

	Patients
	(*N* = 410)
**Demographic characteristics**	
**Sex, n (%)**	
Female	390 (95.1)
Male	20 (4.9)
**Age at diagnosis, mean ± SD [median]**	52 (12.6) [50.0]
18–44 years	137 (33.4)
45–64 years	201 (49.0)
65+ years	72 (17.6)
**Age at diagnosis, categories according to Wood et al.**^a^	
< 40 years	70 (17.1)
40–49 years	128 (31.2)
50–59 years	91 (22.2)
60–69 years	85 (20.7)
≥ 70 years	36 (8.8)
**Race, n (%)**	
White	302 (73.7)
Black or African American	69 (16.8)
Asian	27 (6.6)
Native Hawaiian or Other Pacific Islander	1 (0.2)
Other	11 (2.7)
**Ethnicity, n (%)**	
Hispanic/Latino	43 (10.5)
Non-Hispanic/Latino	354 (86.3)
Unknown	13 (3.2)
**Ashkenazi Jewish ancestry, n (%)**	
Yes	78 (19.0)
No	313 (76.3)
Unknown	19 (4.6)
**Breast cancer pathology**	
**Cancer stage at diagnosis, n (%)**	
Stage 0-III (non-metastatic)	286 (69.8)
Stage IV (metastatic)	124 (30.2)
**ER status, n (%)**	
Positive	244 (59.5)
Negative	166 (40.5)
**PR status, n (%)**	
Positive	206 (50.2)
Negative	204 (49.8)
**HER2 status, n (%)**	
Positive	73 (17.8)
Negative	337 (82.2)
**Triple negative, n (%)**	126 (30.7)
**Nottingham combined histologic grade, n (%)**	
GX (undetermined grade)	2 (0.5)
G1	43 (10.5)
G2	171 (41.7)
G3	192 (46.8)
Unknown	2 (0.5)
**Additional primary breast cancer, n (%)**	
Yes	27 (6.6)
No	382 (93.2)
Unknown	1 (0.2)
**Additional clinical information assessed in female patients**	(*N* = 390)
**Treated for other cancer**^b^**, n (%)**	
Yes, Ovarian carcinoma	18 (4.6)
Yes, Other	10 (2.6)
No	362 (92.8)
**Postmenopausal at diagnosis, n (%)**	
Yes	185 (47.4)
No	201 (51.5)
Unknown	4 (1.0)

***Abbreviations*:***BRCA* Breast Cancer Gene, *ER* Estrogen Receptor, *HER2* Human Epidermal Growth Factor Receptor 2, *NCCN* National Comprehensive Cancer Network, *PR* Progesterone Receptor, *SD* Standard Deviation

^a^Quality of Cancer Family History and Referral for Genetic Counseling and Testing Among Oncology Practices: A Pilot Test of Quality Measures As Part of the American Society of Clinical Oncology Quality Oncology Practice Initiative. Marie E. Wood, Pamela Kadlubek, Trang H. Pham, Dana S. Wollins, Karen H. Lu, Jeffrey N. Weitzel, Michael N. Neuss, and Kevin S. Hughes. Journal of Clinical Oncology 2014 32:8, 824–829

^b^Personal history of cancer in males was not collected, as it was not relevant to determine the risk of *BRCA1* and *BRCA2* pathogenic variants as per NCCN guidelines

### *BRCA* testing and variant status

Among the 410 patients included in this study, 384 (94%) were tested for *BRCA1*/*BRCA2* variants; 24 were not tested, and 2 had an unknown testing status (Table 3). For 75% of the 24 patients who were not tested, physicians deemed that genetic testing would have had no impact on medical management (data not shown). The testing rate was 100% among female patients with at least one close blood relative with a *BRCA* pathogenic variant (*n* = 68), female patients with a diagnosis of breast cancer before age 50 who had an additional primary breast cancer (*n* = 12), and female patients with a personal history of ovarian cancer (*n* = 18). Testing rates over the study period were assessed by year, and tended to range from 88 to 95% from 2013 to 2017, suggesting there was no major change in testing rates over the study period (Additional file 1).

**Table 3:** Testing for *BRCA* Pathogenic Variants in Accordance with NCCN Guidelines^a^ in All Patients

	Total in risk group, n	Patients tested^b^, n (%)	Genetic testing result^c^, n (%)					
			No Pathogenic Variant	Any Pathogenic Variants	***BRCA1*** Pathogenic Variant	***BRCA2*** Pathogenic variants	***BRCA1*** and ***BRCA2*** Pathogenic Variants	Unknown
**All Patients**								
**Total patients included in the study**	410	384 (93.7)	–	–	–	–	–	–
**Risk groups based on NCCN guidelines**^d^								
**Personal history of male breast cancer**	20	18 (90.0)	12 (66.7)	6 (33.3)	4 (22.2)	2 (11.1)	0 (0.0)	0 (0.0)
**Women with at least one close blood relative with a*BRCA1*or*BRCA2*pathogenic variant**	68	68 (100.0)	15 (22.1)	53 (77.9)	34 (50.0)	14 (20.6)	5 (7.4)	0 (0.0)
**Women diagnosed with breast cancer at age ≤ 45**	150	141 (94.0)	95 (67.4)	43 (30.5)	31 (22.0)	8 (5.7)	4 (2.8)	3 (2.1)
**Women diagnosed at age ≤ 50 with**								
An additional primary breast cancer	12	12 (100.0)	4 (33.3)	6 (50.0)	4 (33.3)	2 (16.7)	0 (0.0)	2 (16.7)
At least one close blood relative^e^ with breast cancer at any age	115	109 (94.8)	60 (55.0)	48 (44.0)	34 (31.2)	10 (9.2)	4 (3.7)	1 (0.9)
At least one close blood relative with prostate cancer (Gleason score ≥ 7)	38	33 (86.8)	21 (63.6)	12 (36.4)	8 (24.2)	4 (12.1)	0 (0.0)	0 (0.0)
**Women diagnosed at age ≤ 60 years with**								
Triple negative breast cancer	102	98 (96.1)	58 (59.2)	39 (39.8)	33 (33.7)	4 (4.1)	2 (2.0)	1 (1.0)
**Women diagnosed at any age with**								
At least one close blood relative with breast cancer diagnosed at age ≤ 50 years	148	139 (93.9)	84 (60.4)	54 (38.8)	38 (27.3)	12 (8.6)	4 (2.9)	1 (0.7)
At least two close blood relatives on the same side of the family with breast cancer at any age	164	152 (92.7)	93 (61.2)	57 (37.5)	39 (25.7)	13 (8.6)	5 (3.3)	2 (1.3)
At least one close blood relative with ovarian cancer at any age	116	108 (93.1)	65 (60.2)	42 (38.9)	28 (25.9)	12 (11.1)	2 (1.9)	1 (0.9)
At least two close blood relatives on the same side of the family with pancreatic and/or prostate cancer (Gleason score ≥ 7) at any age	59	54 (91.5)	42 (77.8)	12 (22.2)	9 (16.7)	3 (5.6)	0 (0.0)	0 (0.0)
At least one close male blood relative with breast cancer	18	14 (77.8)	8 (57.1)	6 (42.9)	4 (28.6)	2 (14.3)	0 (0.0)	0 (0.0)
Ashkenazi Jewish or ethnic groups associated with founder mutations	74	72 (97.3)	41 (56.9)	31 (43.1)	19 (26.4)	10 (13.9)	2 (2.8)	0 (0.0)
**Women with a personal history of ovarian cancer**	18	18 (100.0)	7 (38.9)	11 (61.1)	7 (38.9)	3 (16.7)	1 (5.6)	0 (0.0)

***Abbreviations*:***BRCA* Breast Cancer Gene, *NCCN* National Comprehensive Cancer Network

^a^ National Comprehensive Cancer Network. Genetic/Familial High-Risk Assessment: Breast and Ovarian. *BRCA*1/2 Testing Criteria.

^b^ The proportion was calculated out of number of patients in each risk group.

^**c**^ The proportion was calculated out of number of patients who were tested in each risk group.

^d^ Patients can be in more than one risk group based on their personal and family cancer history.

^e^ Close blood relative was defined as mother, father, sisters, brothers, daughters, sons, grandmothers, grandfathers, granddaughters, grandsons, half-siblings, aunts, uncles, nieces, nephews, great-grandmothers, great-grandfathers, great-granddaughters, great-grandsons, great-aunts, great-uncles, and first cousins.

Female patients with male-relative related risk factors had slightly lower testing rates. For example, female patients diagnosed at age ≤ 50 years with at least one close blood relative with prostate cancer (Gleason score ≥ 7), had a lower testing rate of 87% and female patients with at least one close male blood relative with breast cancer had the lowest testing rate of 78% (Table 3). Testing rates were also stratified by year to assess trends over the course of the study period. Due to the small sample size for each of the high risk groups in each year, no clear trends were discernable (Additional file 1).

The proportion of patients who tested positive for a *BRCA* pathogenic variant in various risk groups ranged from 22 to 78% (Table 3). Among the 68 patients who had at least one close blood relative with a known *BRCA*1/2 pathogenic variant, 53 (78%) had a positive result, whereas 62 out of the remaining 316 patients (20%) had a positive result.

Almost all (97%) metastatic and triple negative breast cancer patients were tested for *BRCA* pathogenic variants, and 52% tested positive (Table 4). Among patients with metastatic and HER2(−) breast cancer, 93% underwent *BRCA* testing, of whom 40% tested positive for a *BRCA* pathogenic variant. The testing rate for *BRCA* pathogenic variants in African American patients was slightly lower (91%) than for the overall population. In patients treated in practices with a genetic counselor, 95% were tested for *BRCA* pathogenic variants, compared to 91% of patients treated in practices without a genetic counselor (Table 4).

**Table 4:** Testing for *BRCA* Pathogenic Variants by Patient and Physician Characteristics

	N Patients	Patients tested, n (%)	Patients with any pathogenic variant^a^, n (%)
**All patients**			
Total patients included in the study	410	384 (93.7)	115 (29.9)
**Race**			
White	302	284 (94.0)	94 (33.1)
Black or African American	69	63 (91.3)	15 (23.8)
Asian	27	26 (96.3)	4 (15.4)
Native Hawaiian	1	0 (0.0)	N/A
Other	11	11 (100.0)	2 (18.2)
**Cancer stage**			
Metastatic breast cancer	124	114 (91.9)	42 (36.8)
Early breast cancer	286	270 (94.4)	73 (27.0)
**Clinical characteristics**			
Metastatic and triple negative breast cancer	34	33 (97.1)	17 (51.5)
Metastatic and HER2(−) breast cancer	105	98 (93.3)	39 (39.8)
Triple negative breast cancer	126	121 (96.0)	50 (41.3)
**Genetic counselor**			
Yes	270	256 (94.8)	82 (32.0)
No	140	128 (91.4)	33 (25.8)

***Abbreviations*:***BRCA* Breast Cancer Gene, *HER2* Human Epidermal Growth Factor Receptor 2

^a^The proportion was calculated out of number of patients tested within each category.

Solo practitioners and physicians in small private community practices (1–5 physicians) had the lowest *BRCA* testing rate (92%) (Additional file 1). Additionally, the testing rate was lower for physicians with a greater number of years of experience (21 or more years of experience: 90%) compared to physicians with fewer years of experience (0–11 years of experience: 99%), and for those who treated ≤50 breast cancer patients in the past year (88%) compared to physicians who treated over 50 patients (> 92%).

## Conclusions

This study demonstrates high rates of *BRCA* testing among recently diagnosed breast cancer patients at high risk of hereditary disease, treated by community-based oncologists who elected to participate in this study. Certain risk groups, such as female patients with male-relative related risk factors, had a lower testing rate compared to others, such as patients with a family history of *BRCA* pathogenic variants.

The overall *BRCA* testing rate of 94% in this study was higher than, though generally in line with, testing rates found in similar studies conducted since 2013. A 2016 study by Rosenberg et al. of 897 women aged ≤ 40 years at breast cancer diagnosis treated in an academic or community medical center reported a testing rate of 87% by 1 year post-diagnosis [10]. The investigators noted that the frequency of *BRCA* testing among women diagnosed with breast cancer between 2006 and 2013 increased from 77 to 95% [10]. Similarly, Chen et al. found that the *BRCA* testing rate increased by 57% in 2013, compared to 11% average annual increases during the preceding 3 years [11]. The current study found that testing rates from 2013 to 2017 remained relatively stable. A recent analysis by Katz et al. conducted in breast cancer patients at an elevated risk of pathogenic variant and treated in a community-based setting, found that approximately half received genetic testing, a lower figure than reported in this study [12]. Unlike this study, the previous analysis by Katz et al. did not include any patients with metastatic breast cancer, and did not report how many women had TNBC. An overall positive test result was observed in 78% of patients who had at least one close blood relative with a known *BRCA* pathogenic variant, and in 20% of patients with no close blood relatives with a *BRCA* pathogenic variant. High positive rates of about 40% were also observed in patients with TNBC (who were oversampled) and metastatic and HER2(−) breast cancer. Katz et al. observed variants of uncertain significance or pathogenic variant in 22.7% of tested women. In this study, however, physicians were not asked how they characterized a result of variants of uncertain significance on the CRF. High rates of pathogenic variants among TNBC patients have previously been reported [13, 14].

This study was designed to identify the *BRCA* testing rate among patients meeting NCCN criteria for testing, thus we would expect to estimate a higher testing rate than for the general population of breast cancer patients. Additionally, the high testing rates observed in this study are applicable to the physicians who elected to contribute data. These physicians had extensive clinical experience, inferred by the fact that they treated an average of 154 breast cancer patients in the previous year, and that they had an average of 16 years of clinical practice. A large proportion also had a genetic counselor within his or her practice. All patients included in this study had documented family history of cancers, which is key to identifying those at a greater risk of hereditary breast cancer. The *BRCA* testing rate may be lower in patients with unknown, or without documented, family history. Wood et al. found that oncologists participating in the 2011 American Society of Clinical Oncology (ASCO) Quality Oncology Practice Initiative (QOPI), documented a complete family history for less than 40% of the breast cancer patients they treated [8]. It is important to promote education of elements of a complete documented family history, such as family history of cancers with age of diagnosis in first- and second-degree relatives. Lastly, this study required the availability of HER2 receptor status, which may have excluded patients with ductal carcinoma in situ (DCIS) as these patients are frequently not tested for HER2. Conclusions regarding testing rates cannot be applied to these patients.

Several key milestones in 2013 may have impacted *BRCA* testing rates, including the expanded availability of next-generation sequencing (NGS) technology, the revocation of the Myriad *BRCA* test patent by the US Supreme Court that resulted in a greater number of laboratories offering the test, and the publication of a celebrity editorial and resulting increased publicity around *BRCA* testing [15]. Previous studies have estimated a 37–64% relative increase in the rate of *BRCA* testing immediately following the publication of the celebrity editorial [16, 17]. Furthermore, the Affordable Care Act passed in 2010 gave the US population greater access to healthcare in addition to full coverage of *BRCA* testing for many individuals who were not previously eligible. This study focused on patients diagnosed with breast cancer during and after 2013, therefore, the high testing rate may be explained by changes in clinical practice and societal attitudes that encouraged *BRCA* testing among high risk patients.

Among community-based physicians who had extensive clinical experience and the majority of whom had a genetic counselor as part of their practice, the reported rate of *BRCA* testing was high. These findings are encouraging as targeted breast cancer treatments for patients with *BRCA* pathogenic variants, such as olaparib, have been approved. Among metastatic and HER2(−) breast cancer patients with a germline *BRCA* pathogenic variant, olaparib was found to significantly prolong progression-free survival compared with standard of care chemotherapy in a phase III clinical trial [18]. Talazoparib, another targeted treatment, was also recently found to improve progression-free survival over standard chemotherapy among advanced breast cancer patients with a germline *BRCA* pathogenic variant [19].

Our study demonstrates that a high level of compliance to guidelines in a community setting is possible with a delivery model for genetic counseling and testing. Steps in this direction can help improve treatment for breast cancer patients. Additional training may also be helpful, for example, although not assessed in this study, previous research has indicated that physicians who were trained in clinical genetics in medical school or through continuing medical education (CME) were more likely to have ordered or referred a patient for genetic testing [20]. When physicians maintain high testing rates in accordance with NCCN guidelines, patients identified to have a *BRCA* pathogenic variant have the option of using a targeted therapy, such as olaparib or talazoparib. In fact, multi-gene panel testing utilizing next generation sequencing has enabled physicians to identify pathogenic variants in genes other than *BRCA* 1 and 2, and further research is needed to determine the rates of multi-gene panel testing and adherence to NCCN recommendation for additional high-risk genes (though this was not within the scope of the current study). Improvements in *BRCA* 1 and 2 testing rates are needed for patients with metastatic breast cancer, with risk factors related to male blood relatives, and patients of African American heritage, to ensure that all breast cancer patients can make informed treatment decisions.

## Supplementary information

**Additional file 1 Appendix Table 1**. Patient Characteristics in Metastatic and Triple Negative Patients/Metastatic and HER2(−) Patients **Appendix Table 2**. Proportion of Patients Tested for *BRCA* Pathogenic Variants by Physician Characteristics** Appendix Table 3**. Proportion Tested for *BRCA* Pathogenic Variants in Accordance with NCCN Guidelines by Year of Breast Cancer Diagnosis.

## Data Availability

The datasets used and/or analyzed during the current study are available from the corresponding author on reasonable request.

## Abbreviations

ASCO: American Society of Clinical Oncology
*BRCA*: BReast CAncer gene
eCRF: Electronic case report form
ER: Estrogen receptor
HER2: Human Epidermal Growth Factor Receptor 2
HR: Hormone receptor
IRB: Institutional review board
NCCN: National Comprehensive Cancer Network
NGS: Next-generation sequencing
PARP: Poly (ADP-ribose) polymerase
PR: Progesterone receptor
QOPI: Quality Oncology Practice Initiative
TNBC: Triple negative breast cancer
