# Supplementary material for: Adherence to National Comprehensive Cancer Network Guidelines for BRCA testing among high risk breast Cancer patients: a retrospective chart review study
Source: Hered Cancer Clin Pract. 2020 Jun 6;18:13. doi: 10.1186/s13053-020-00144-z (PMC7275608; doi:10.1186/s13053-020-00144-z)
Supplement: Supplementary file 1 — Additional file 1 Appendix Table 1. Patient Characteristics in Metastatic and Triple Negative Patients/Metastatic and HER2(−) Patients Appendix Table 2. Proportion of Patients Tested for BRCA Pathogenic Variants by Physician Characteristics Appendix Table 3. Proportion Tested for BRCA Pathogenic Variants in Accordance with NCCN Guidelines by Year of Breast Cancer Diagnosis. [file 13053_2020_144_MOESM1_ESM.docx]

# Appendix

## Appendix 1

Study inclusion criteria

In order to be eligible for inclusion in the study, patients were required to meet the following inclusion criteria:

- Have a confirmed diagnosis of initial breast cancer between January 2013 and October 2017 with an accompanying pathology report such that hormone receptor (HR) status (i.e., estrogen receptor [ER], progesterone receptor [PR]) and Human Epidermal Growth Factor Receptor 2 (HER2) status were known
- Aged 18 years or older at initial breast cancer diagnosis
- Have information on family history of cancer
- Have an increased hereditary risk of breast cancer as indicated by:
  - Breast cancer diagnosed at age ≤45 years; OR
  - Male breast cancer; OR
  - Ashkenazi Jewish ancestry; OR
  - Personal or family history of ovarian cancer; OR
  - More than one primary breast cancer, and the first was diagnosed at age < 50 years; OR
  - Personal history of triple negative breast cancer at age ≤60 years; OR
  - Family history of a positive cancer gene mutation found by genetic testing; OR
  - Family history (up to 3^rd^ degree relatives) of at least one relative with breast cancer diagnosed at age ≤50 years, or male breast cancer; OR
  - Family history (up to 3^rd^ degree relatives) of at least two relatives on the same side of the family with breast cancer, pancreatic cancer, or prostate cancer (any age for these diagnoses)

These criteria to identify high hereditary risk were developed based on the National Comprehensive Cancer Network (NCCN) testing guidelines and criteria commonly used to identify high risk patients in clinical practice.

NCCN high risk groups

Breast cancer patients were categorized into the following high risk groups as defined in NCCN guidelines:

- Men with a personal history of breast cancer
- Women with at least one close blood relative with a *BRCA*1 or *BRCA*2 pathogenic variant
- Women diagnosed with breast cancer at age ≤45
- Women diagnosed at age ≤50 with
  - An additional primary breast cancer
  - At least one close blood relative with breast cancer at any age
  - At least one close blood relative with prostate cancer (Gleason score ≥7)
- Women diagnosed at age ≤60 with
  - Triple-negative breast cancer
- Women diagnosed at any age with
  - At least one close blood relative with breast cancer diagnosed at age ≤50 years
  - At least two close blood relatives on the same side of the family with breast cancer at any age
  - At least one close blood relative with ovarian cancer at any age
  - At least two close blood relatives on the same side of the family with pancreatic and/or prostate cancer (Gleason score ≥7) at any age
  - At least one close male blood relative with breast cancer
  - Ashkenazi Jewish or ethnic groups associated with founder mutations
- Women with a personal history of ovarian cancer,

Close blood relative was defined as mother, father, sisters, brothers, daughters, sons, grandmothers, grandfathers, granddaughters, grandsons, half-siblings, aunts, uncles, nieces, nephews, great-grandmothers, great-grandfathers, great-granddaughters, great-grandsons, great-aunts, great-uncles, and first cousins.

Note that high-risk groups were described consistently across guidelines released in all years of the study period (i.e., 2013-2017) with only one exception: women diagnosed with breast cancer at age ≤50 years with at least one close blood relative with prostate cancer (Gleason score ≥7) were included in all guidelines with the exception of 2013. However, we do not believe this has a meaningful impact on our results as this group had a testing rate of 100% in 2013 even though they were not included as a high risk category in the 2013 NCCN guidelines (see Appendix Table 3).

## Appendix tables

**Appendix Table 1. Patient Characteristics in Metastatic and Triple Negative Patients/Metastatic and HER2(-) Patients**

|  | **Metastatic and triple negative patients** | **Metastatic and HER2(-) patients** |
| --- | --- | --- |
|  | (N = 34) | (N = 105) |
| **Demographic characteristics** | |  |
| **Sex, n (%)** |  |  |
| Female | 34 (100.0) | 103 (98.1) |
| Male | 0 (0.0) | 2 (1.9) |
| **Age at diagnosis, mean ± SD [median]** | 51 (11.3) [48.5] | 57 (12.7) [57.0] |
| 18-44 years | 11 (32.4) | 21 (20.0) |
| 45-64 years | 18 (52.9) | 53 (50.5) |
| 65+ years | 5 (14.7) | 31 (29.5) |
| **Race, n (%)** |  |  |
| White | 22 (64.7) | 82 (78.1) |
| Black or African American | 11 (32.4) | 18 (17.1) |
| Asian | 1 (2.9) | 5 (4.8) |
| Native Hawaiian or Other Pacific Islander | 0 (0.0) | 0 (0.0) |
| Other | 0 (0.0) | 0 (0.0) |
| **Ethnicity, n (%)** |  |  |
| Hispanic/Latino | 1 (2.9) | 9 (8.6) |
| Non-Hispanic/Latino | 29 (85.3) | 92 (87.6) |
| Unknown | 4 (11.8) | 4 (3.8) |
| **Ashkenazi Jewish ancestry, n (%)** | |  |
| Yes | 5 (14.7) | 30 (28.6) |
| No | 26 (76.5) | 71 (67.6) |
| Unknown | 3 (8.8) | 4 (3.8) |
| **Breast cancer pathology** | |  |
| **ER status, n (%)** |  |  |
| Positive | 0 (0.0) | 65 (61.9) |
| Negative | 34 (100.0) | 40 (38.1) |
| **PR status, n (%)** |  |  |
| Positive | 0 (0.0) | 52 (49.5) |
| Negative | 34 (100.0) | 53 (50.5) |
| **Triple negative, n (%)** | 34 (100.0) | 34 (32.4) |
| **Nottingham combined histologic grade, n (%)** | |  |
| GX (undetermined grade) | 1 (2.9) | 1 (1.0) |
| G1 | 0 (0.0) | 10 (9.5) |
| G2 | 6 (17.6) | 40 (38.1) |
| G3 | 27 (79.4) | 54 (51.4) |
| Unknown | 0 (0.0) | 0 (0.0) |
| **Additional primary breast cancer, n (%)** | |  |
| Yes | 0 (0.0) | 3 (2.9) |
| No | 34 (100.0) | 102 (97.1) |
| Unknown | 0 (0.0) | 0 (0.0) |
| **Additional clinical information assessed in female patients** | (N = 34) | (N = 103) |
| **Treated for other cancer^1^, n (%)** | |  |
| Yes, Ovarian carcinoma | 1 (2.9) | 5 (4.9) |
| Yes, Other | 0 (0.0) | 1 (1.0) |
| No | 33 (97.1) | 97 (94.2) |
| **Postmenopausal at diagnosis, n (%)** | |  |
| Yes | 17 (50.0) | 66 (64.1) |
| No | 17 (50.0) | 35 (34.0) |
| Unknown | 0 (0.0) | 2 (1.9) |

**Abbreviations:** *BRCA*, Breast Cancer Gene; ER, Estrogen Receptor; HER2, Human Epidermal Growth Factor Receptor 2; NCCN, National Comprehensive Cancer Network; PR, Progesterone Receptor; SD, Standard Deviation.

**Note:**

[1] Personal history of cancer in males was not collected, as it was not relevant to determine the risk of *BRCA*1 and *BRCA*2 pathogenic variants as per NCCN guidelines.

**Appendix Table 2. Proportion of Patients Tested for *BRCA* Pathogenic Variants by Physician Characteristics**

|  | **Patients, n** | **Patients tested^1^, n (%)** |
| --- | --- | --- |
|  |  |  |
| **All Patients** |  |  |
| Total patients included in the study | 410 | 384 (93.7) |
| **Practice setting of physician** |  |  |
| Solo practitioner or small private community practice (1-5 physicians) | 100 | 92 (92.0) |
| Medium-sized private community practice (6-10 physicians) | 102 | 98 (96.1) |
| Large private community practice (>10 physicians) or community practice owned by a hospital | 203 | 189 (93.1) |
| Other^2^ | 5 | 5 (100.0) |
| **Years of practice of physician** |  |  |
| Q1: 0 - 11 | 67 | 66 (98.5) |
| Q2: 12 - 14 | 101 | 95 (94.1) |
| Q3: 15 - 20 | 129 | 121 (93.8) |
| Q4: 21 or more | 113 | 102 (90.3) |
| **Number of patients physician treated in the past year** |  |  |
| Q1: 0 - 49 | 91 | 80 (87.9) |
| Q2: 50 - 99 | 103 | 100 (97.1) |
| Q3: 100 - 199 | 89 | 82 (92.1) |
| Q4: 200 or more | 127 | 122 (96.1) |

**Abbreviations:** *BRCA*, Breast Cancer Gene; NCCN, National Comprehensive Cancer Network.

**Notes:**

[1] The proportion was calculated out of number of patients within each category.

[2] Other practice settings include affiliation with academic institutions.

| **Appendix Table 3. Proportion Tested for *BRCA* Pathogenic Variants in Accordance with NCCN Guidelines^1^ by Year of Breast Cancer Diagnosis** | | | | | |
| --- | --- | --- | --- | --- | --- |
|  | **Patients tested by year of diagnosis^2^, n (%)** | | | | |
|  | 2013 | 2014 | 2015 | 2016 | 2017 |
| **Total patients** | 42 (95.5) | 57 (87.7) | 88 (95.7) | 104 (95.4) | 93 (93.0) |
| **Risk groups based on NCCN guidelines^3^** |  |  |  |  |  |
| **Personal history of male breast cancer** | 2 (66.7) | 3 (100.0) | 5 (100.0) | 4 (80.0) | 4 (100.0) |
| **Women with at least one close blood relative with a *BRCA1* or *BRCA2* pathogenic variant** | 6 (100.0) | 4 (100.0) | 16 (100.0) | 23 (100.0) | 19 (100.0) |
| **Women diagnosed with breast cancer at age ≤45** | 14 (100.0) | 18 (94.7) | 27 (90.0) | 41 (95.3) | 41 (93.2) |
| **Women diagnosed at age ≤50 with** |  |  |  |  |  |
| An additional primary breast cancer | 1 (100.0) | 3 (100.0) | 3 (100.0) | 1 (100.0) | 4 (100.0) |
| At least one close blood relative with breast cancer at any age | 10 (100.0) | 18 (90.0) | 26 (92.9) | 31 (93.9) | 24 (100.0) |
| At least one close blood relative with prostate cancer (Gleason score ≥7) | 3 (100.0) | 7 (87.5) | 9 (81.8) | 7 (87.5) | 7 (87.5) |
| **Women diagnosed at age ≤60 years with** |  |  |  |  |  |
| Triple-negative breast cancer | 11 (100.0) | 14 (100.0) | 19 (90.5) | 28 (100.0) | 26 (92.9) |
| **Women diagnosed at any age with** |  |  |  |  |  |
| At least one close blood relative with breast cancer diagnosed at age ≤50 years | 15 (100.0) | 21 (91.3) | 32 (94.1) | 46 (95.8) | 25 (89.3) |
| At least two close blood relatives on the same side of the family with breast cancer at any age | 19 (100.0) | 28 (87.5) | 36 (92.3) | 44 (93.6) | 25 (92.6) |
| At least one close blood relative with ovarian cancer at any age | 14 (100.0) | 14 (77.8) | 28 (96.6) | 32 (91.4) | 20 (100.0) |
| At least two close blood relatives on the same side of the family with pancreatic and/or prostate cancer (Gleason score ≥7) at any age | 7 (87.5) | 16 (94.1) | 11 (84.6) | 15 (93.8) | 5 (100.0) |
| At least one close male blood relative with breast cancer | 1 (100.0) | 2 (66.7) | 3 (75.0) | 1 (33.3) | 7 (100.0) |
| Ashkenazi Jewish or ethnic groups associated with founder mutations | 6 (100.0) | 4 (80.0) | 21 (100.0) | 19 (100.0) | 22 (95.7) |
| **Women with a personal history of ovarian cancer** | 2 (100.0) | 2 (100.0) | 7 (100.0) | 7 (100.0) | 0 (0.0) |
| **Abbreviations:** *BRCA*, Breast Cancer Gene; NCCN, National Comprehensive Cancer Network. | | | | | |
| **Notes:** | | | | | |
| [1] National Comprehensive Cancer Network. Genetic/Familial High-Risk Assessment: Breast and Ovarian. *BRCA*1/2 Testing Criteria | | | | | |
| [2] The proportion was calculated out of number of patients in each risk group. | | | | | |
| [3] Patients can be in more than one NCCN risk group based on their personal and family cancer history. | | | | | |
